# Large intrinsic anomalous Hall effect in SrIrO_3_ induced by magnetic proximity effect

**DOI:** 10.1038/s41467-021-23489-y

**Published:** 2021-06-02

**Authors:** Myoung-Woo Yoo, J. Tornos, A. Sander, Ling-Fang Lin, Narayan Mohanta, A. Peralta, D. Sanchez-Manzano, F. Gallego, D. Haskel, J. W. Freeland, D. J. Keavney, Y. Choi, J. Strempfer, X. Wang, M. Cabero, Hari Babu Vasili, Manuel Valvidares, G. Sanchez-Santolino, J. M. Gonzalez-Calbet, A. Rivera, C. Leon, S. Rosenkranz, M. Bibes, A. Barthelemy, A. Anane, Elbio Dagotto, S. Okamoto, S. G. E. te Velthuis, J. Santamaria, Javier E. Villegas

**Affiliations:** 1 Unité Mixte de Physique, CNRS, Thales, Université Paris-Saclay, Palaiseau, France; 2 GFMC, Dept. Fisica de Materiales, Facultad de Fisica, Universidad Complutense, Madrid, Spain; 3 Department of Physics and Astronomy, University of Tennessee, Knoxville, TN USA; 4 School of Physics, Southeast University, Nanjing, China; 5 Materials Science and Technology Division, Oak Ridge National Laboratory, Oak Ridge, TN USA; 6 Advanced Photon Source Argonne National Laboratory, Lemont, IL USA; 7 Department of Physics, Bryn Mawr College, Bryn Mawr, PA USA; 8 IMDEA Nanoscience Campus Universidad Autonoma, Cantoblanco, Spain; 9 Centro Nacional de Microscopia Electronica, Universidad Complutense, Madrid, Spain; 10 CELLS-ALBA Synchrotron Radiation Facility, Cerdanyola del Valles, Spain; 11 Department Quimica Inorganica, Facultad de Quimica, Universidad Complutense, Madrid, Spain; 12 Materials Science Division, Argonne National Laboratory, Lemont, IL USA

**Keywords:** Electronic properties and materials, Surfaces, interfaces and thin films

## Abstract

The anomalous Hall effect (AHE) is an intriguing transport phenomenon occurring typically in ferromagnets as a consequence of broken time reversal symmetry and spin-orbit interaction. It can be caused by two microscopically distinct mechanisms, namely, by skew or side-jump scattering due to chiral features of the disorder scattering, or by an intrinsic contribution directly linked to the topological properties of the Bloch states. Here we show that the AHE can be artificially engineered in materials in which it is originally absent by combining the effects of symmetry breaking, spin orbit interaction and proximity-induced magnetism. In particular, we find a strikingly large AHE that emerges at the interface between a ferromagnetic manganite (La_0.7_Sr_0.3_MnO_3_) and a semimetallic iridate (SrIrO_3_). It is intrinsic and originates in the proximity-induced magnetism present in the narrow bands of strong spin-orbit coupling material SrIrO_3_, which yields values of anomalous Hall conductivity and Hall angle as high as those observed in bulk transition-metal ferromagnets. These results demonstrate the interplay between correlated electron physics and topological phenomena at interfaces between 3*d* ferromagnets and strong spin-orbit coupling 5*d* oxides and trace an exciting path towards future topological spintronics at oxide interfaces.

## Abstract (web summary)

The anomalous Hall effect (AHE) occurs in ferromagnets caused by intrinsic and extrinsic mechanisms. Here, Yoo *et al*. report large anomalous Hall conductivity and Hall angle at the interface between a ferromagnet La_0.7_Sr_0.3_MnO_3_ and a semimetallic SrIrO_3_, due to the interplay between correlated physics and topological phenomena.

## Introduction

The Hall resistivity of ferromagnets has been shown to follow the empirical relation^1^ ${\rho }_{{xy}}={R}_{0}{H}_{z}+\Delta {\rho }_{{xy}}^{\text{AHE}}{M}_{z}$. The first term is the ordinary Hall resistivity, and the normal Hall parameter R_0_ depends basically on the carrier density. The second term scales with the sample magnetization ${M}_{z}$ and is the anomalous Hall effect (AHE), which is characterized by the anomalous Hall coefficient $\Delta {\rho }_{{xy}}^{\text{AHE}}$. The Hall resistivity can be written in terms of the Hall conductivity ${\sigma }_{{xy}}$ and the longitudinal resistivity ${\rho }_{{xx}}$ by inverting the conductivity tensor, which yields ${\rho }_{{xy}}={\sigma }_{{xy}}{{\rho }_{{xx}}}^{2}$. In general, ${\sigma }_{{xy}}$ is the sum of different contributions (ordinary and anomalous), which reflects the additivity of the Hall currents. This is especially useful when studying the anomalous Hall conductivity, ${\sigma }_{{xy}}^{{{\mathrm{AHE}}}}$, which results from broken time-reversal symmetry in the presence of spin-orbit interaction, and may contain itself two different contributions^1^. On the one hand, spin-orbit (skew) scattering produces the so-called “extrinsic” contribution, ${\sigma }_{{xy}}^{{{\mathrm{AHEskew}}}}$, which depends linearly on the scattering rate, and thus yields a Hall resistivity directly proportional to the longitudinal resistivity, $\Delta {\rho }_{{xy}}^{\text{AHE}}\propto$ ${\rho }_{{xx}}$. On the other hand, the so-called “intrinsic” contribution ${\sigma }_{{xy}}^{{{\mathrm{AHE}}}{{\mathrm{int}}}}$ is independent of the scattering rate and thus shows scaling of the form $\Delta {\rho }_{{xy}}^{{\rm{AHE}}}\propto {\rho }_{{xx}}^{2}$. The intrinsic contribution is directly connected to the topology of the electronic bands^2^. In fact, ${\sigma }_{{xy}}^{{{\mathrm{AHE}}}{{\mathrm{int}}}}$can be expressed as the integral of the Berry phase curvature over occupied states in the Brillouin zone^1,3,4^.

Broken time-reversal symmetry is a requisite for AHE, and thus the intrinsic AHE is typically observed in ferromagnets^1^ and, very recently, also in non-collinear antiferromagnets with small ferromagnetic moments^5,6^. However, here we show that a large intrinsic AHE can be induced in a non-magnetic 5*d* oxide by proximity with a ferromagnetic 3*d* oxide. This type of 3*d*/5*d* oxide interface has already proven an exciting playground to explore novel contributions to the intrinsic Hall effect which are related to the topological properties of correlated electrons^7,8^. In this work we find that the AHE induced in the 5*d* oxide is very strong, has an opposite sign to that in the 3*d* ferromagnet, and it emerges due to magnetic proximity interaction. This AHE is triggered by the topological properties of the electronic states in the 5*d* oxide.

Our study focuses on interfacial magnetism and the Hall effect in La_0.3_Sr_0.7_MnO_3_ (LSMO)/SrIrO_3_ (SIO or SIO-113) heterostructures. LSMO is a 3*d* half-metallic ferromagnet with strongly correlated electrons^9^. Manganites are double exchange systems where itinerant ${e}_{g}$ electron spins are coupled by the Hund interaction to the localized ${t}_{2g}$ spins. AHE in manganites is known to result from non-coplanar spin configurations of the localized ${t}_{2g}$ manifolds which acquire a scalar spin chirality (real space Berry phase) acting as a virtual magnetic field in real space yielding an intrinsic (real space) contribution to the AHE^10,11^ and eventually also large topological Hall effect^12^. SIO-113, a strong spin-orbit material with a paramagnetic semimetallic ground-state, has been the subject of great attention. The perovskite form of SrIrO_3_, metastable in bulk samples, is stabilized by epitaxial strain in thin films^13–20^. Ir is in a 4+ oxidation state, and the large octahedral crystal field stabilizes the five 5*d* electrons in a ${t}_{2g}^{5}$ low spin configuration^21,22^. Angle-resolved photoemission spectroscopy has shown that the exotic semi-metallic ground state results from the heavy hole-like and light electron-like bands crossing the Fermi level^19^. The strong spin-orbit interaction (0.3–0.4 eV/atom) couples the electronic structure to a complex pattern of octahedral rotations resulting in extremely narrow bands with a bandwidth in the range of 0.3 eV where the Coulomb repulsion (with a comparable energy scale of 0.3 to 0.4 eV) results in electron correlations triggering a metal-insulator transition (MIT) in ultrathin films^23,24^. Notably, Coulomb repulsion, spin-orbit interaction, and bandwidth with comparable energy scales conspire to establish novel electronic ground states.

## Results

### Sample growth and structure

**Fig. 1:**
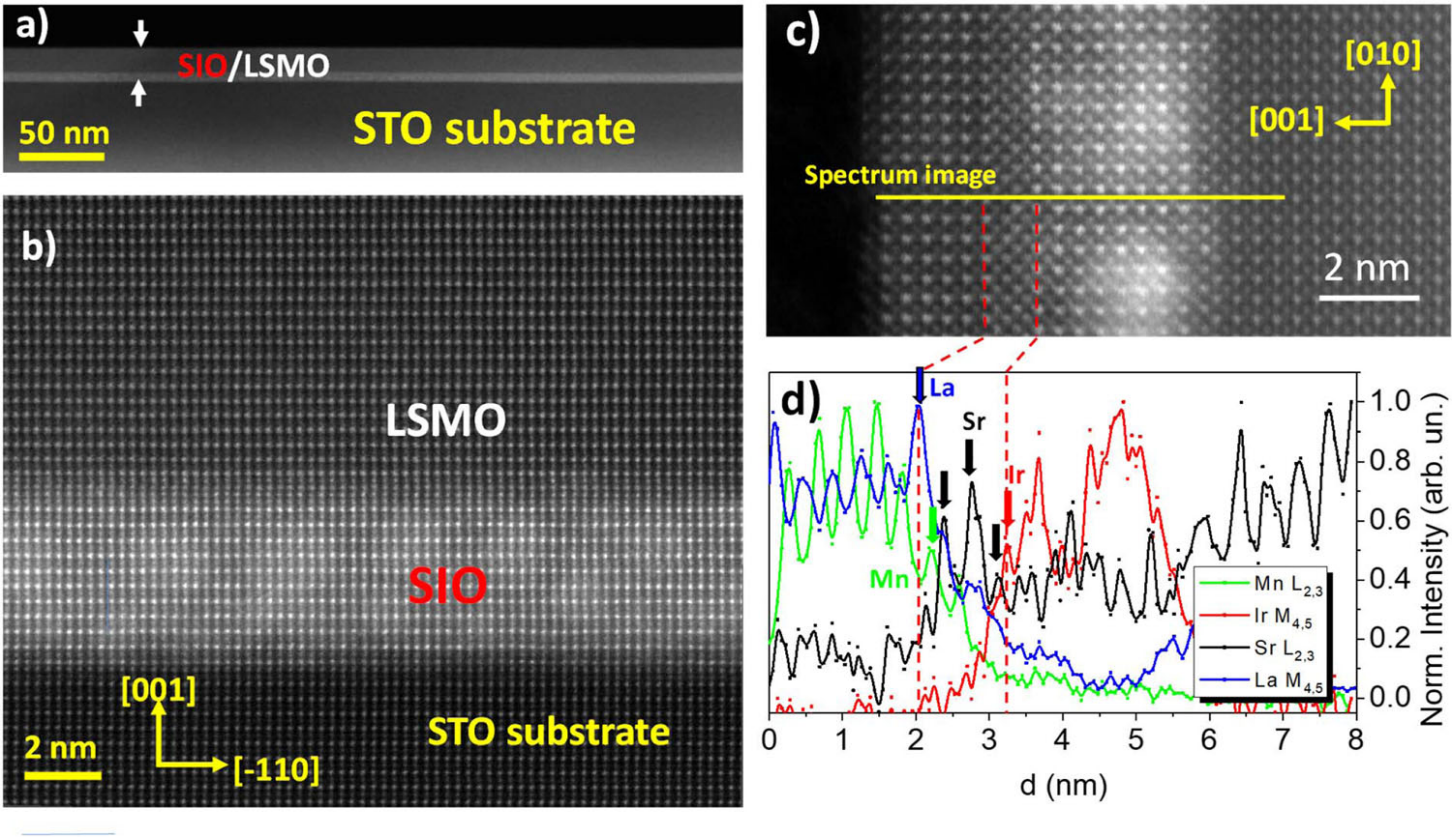
Structure and chemistry of the interfaces. **a** Low magnification HAADF image of an LSMO (15 nm)/SIO (4.4 nm) sample. **b** High-resolution image of the [110] [001] SIO/LSMO interface. **c** High-resolution image of the [010] [001] LSMO (2.4 nm)/SIO (3 nm) interface and corresponding normalized intensity profile of the Mn L_2,3_ (green symbols), La M_4,5_ (blue symbols), and Ir M_4,5_ (red symbols) and Sr L_2,3_ (black symbols), of an EELS line scan acquired across the LSMO/SIO/STO interfaces.

Samples were epitaxially grown on (001) oriented SrTiO_3_ substrates in a sputtering apparatus with a pure oxygen atmosphere at high pressures (3 mbar) and elevated temperatures (650 °C for SIO and 900 °C for LSMO). The structure and chemistry of the interfaces were examined by aberration-corrected scanning transmission electron microscopy (STEM) combined with electron energy loss spectroscopy (EELS) measurements. Figures 1a and 1b display high angle annular dark-field (HAADF) images of a bilayer of 4.4 nm of SIO and 15 nm of LSMO. The bilayers grow flat and coherent over long lateral distances, as shown in Fig. 1a. The HAADF image of Fig. 1b has been obtained from a cross-section sample thinned in the [110]/[001] plane. Since contrast scales with atomic number, the heavier Ir atoms at the B site of the perovskite (IrO_2_ planes) appear brighter in the SIO, while at the LSMO side La/Sr (A site of the perovskite) is brighter. Looking at the interface a contrast anomaly can be clearly recognized, which evidences an alteration of the layer sequence at the SIO/LSMO interface. This can be further examined by combined HAADF image and EELS in a cross-section [010]/[001] sample. The spectrum image of Fig. 1c corresponds to a sample SIO (3 nm)/LSMO (2.4 nm). In this image, the heavier Ir atoms at the B site of the perovskite (IrO_2_ planes) in SIO are laterally displaced with respect to the brighter La/Sr columns (A site of the perovskite) at the LSMO side. Figure 1c shows the Mn L_2,3_, La M_4,5_, and Sr L_2,3_ and Ir M_4,5_ integrated signals of an EELS line scan perpendicular to the interface as shown by the yellow line in Fig. 1c. Comparing the decay of the La M_4,5_ and Mn L_2,3_ signals towards the interface confirms that the SIO/LSMO interfacial plane is (La, Sr)/O. There is an extra SrO plane at the interface resulting in a LaSrO/SrO/SrO/IrO_2_ layer sequence (Sr planes are marked with black arrows in Fig. 1c). The two consecutive SrO planes are displaced laterally with respect to each other as in a rock salt structure. This interface reconstruction has not been observed in other manganite iridates superlattices^25–27^ grown by pulsed laser deposition (PLD), although it happens often at interfaces between perovskites involving members of the Ruddlesden-Popper series^28–30^. As shown below, and similarly as in other manganite-based heterostructures^30^, the two interfacial SrO planes will have deep implications on the interfacial magnetic properties of the heterostructures.

### Transport characterization

**Fig. 2:**
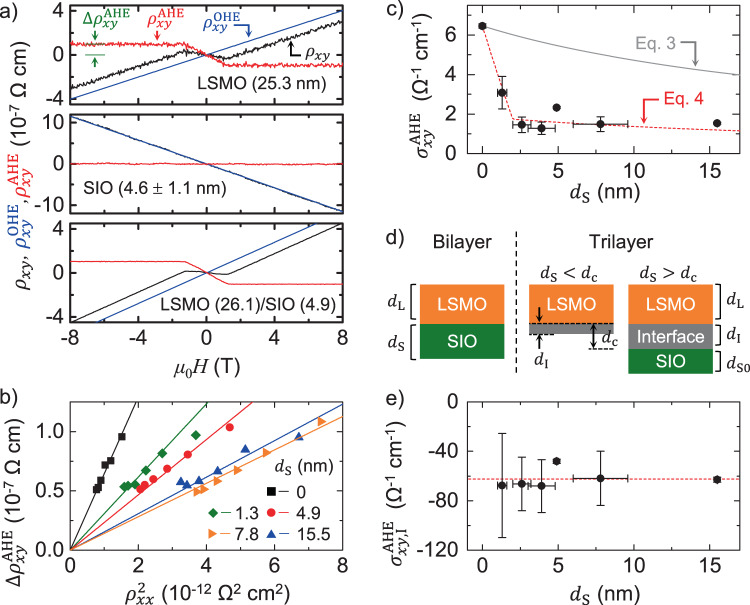
Hall effect measurement and analysis. **a** Transverse Hall resistivity ${\rho }_{{xy}}$ vs. out of the plane magnetic field, *H*_*z*_, (black lines) of La_0.7_Sr_0.3_MnO_3_ 25.3 nm (upper panel) and SrIrO_3_ 4.6 ± 1.1 nm (middle panel) thin films and an LSMO (26.1 nm)/SIO(4.9 nm) bilayer (lower panel) measured at *T* = 100 K. Ordinary Hall resistivity ${\rho }_{{xy}}^{\text{OHE}}$(blue lines) and anomalous Hall resistivity, ${\rho }_{{xy}}^{\text{AHE}}$(red lines) have been separated. $\Delta {\rho }_{{xy}}^{\text{AHE}}$ indicates the value of ${\rho }_{{xy}}^{\text{AHE}}$ at magnetic saturation (green arrow) **b** $\Delta {\rho }_{{xy}}^{\text{AHE}}$ vs. ${\rho }_{{xx}}^{2}$ of La_0.7_Sr_0.3_MnO_3_/SrIrO_3_ bilayers for different thicknesses ${d}_{\text{S}}$of SrIrO_3_ (symbols). $\Delta {\rho }_{{xy}}^{\text{AHE}}$ and ${\rho }_{{xx}}$ are measured at *T* = 20, 35, 50, 65, 80, and 100 K for each ${d}_{\text{S}}$. Lines are linear fits to Eq. 1**c** AHE conductivity, ${\sigma }_{{xy}}^{\text{AHE}}$, vs. thickness of the SrIrO_3_ layer, ${d}_{\text{S}}$ of bilayer samples. The symbols are experimental values of the Hall conductivity obtained from linear fitting curves in Fig. 2b. The gray solid line indicates the calculated ${\sigma }_{{xy}}^{\text{AHE}}$ using a bilayer model (Eq. 3). To calculate ${\sigma }_{{xy}}^{\text{AHE}}$, we use ${d}_{{\rm{L}}}$ = 27.3 nm which is the average value of ${d}_{{\rm{L}}}$ of the different samples. The red dashed line is the calculated ${\sigma }_{{xy}}^{\text{AHE}}$ using a trilayer model (Eq. 4). For the calculation, ${d}_{{\rm{c}}}$ = 2 nm and ${\sigma }_{{xy},\text{I}}^{\text{AHE}}=-62.5{\Omega }^{-1}\bullet {{\rm{cm}}}^{-1}$ are chosen. **d** Sketch illustrating the bilayer and the trilayer models used to fit Hall data (see text). **e** Calculated AHE conductivity of the interface layer, ${\sigma }_{{xy},\text{I}}^{\text{AHE}}$, as a function of ${d}_{{\rm{S}}}$ using Eq. 5. For the calculation, we used ${d}_{{\rm{c}}}$ = 2 nm based on the XMCD measurements. The red dashed line indicate ${\sigma }_{{xy},\text{I}}^{\text{AHE}}\simeq -62.5{\Omega }^{-1}\bullet c{{\rm{m}}}^{-1}$ which is a mean value when ${d}_{{\rm{S}}}$ > 2 nm.

We first studied the intrinsic Hall effect in LSMO and SIO, by measuring the Hall resistivity ${\rho }_{{xy}}$ at low temperature (*T* ≤ 100 K) in individual LSMO (thickness ${d}_{\text{L}}$ = 25.3 nm) and SIO films (${d}_{\text{S}}$ = 4.6 ± 1.1 nm). The measured ${\rho }_{{xy}}$ includes both the contributions of the ordinary Hall effect (OHE) ${\rho }_{{xy}}^{{{\mathrm{OHE}}}}$ and the anomalous Hall one ${\rho }_{{xy}}^{\text{AHE}}$, that is, ${\rho }_{{xy}}={\rho }_{{xy}}^{\text{OHE}}+{\rho }_{{xy}}^{\text{AHE}}$, where ${\rho }_{{xy}}^{\text{OHE}}\propto {H}_{z}$ and ${\rho }_{{xy}}^{\text{AHE}}\propto {M}_{z}$ with ${H}_{z}$ the magnetic field and ${M}_{z}$ the magnetization perpendicular to the film plane. Note that ${\rho }_{{xy}}^{\text{AHE}}$ becomes constant once ${M}_{z}$is saturated by the applied field ${H}_{z}$. This allows determining ${\rho }_{{xy}}^{\text{OHE}}$ and ${\rho }_{{xy}}^{\text{AHE}}$ from the measured ${\rho }_{{xy}}$, as shown in the example of Fig. 2a for plain LSMO films. The lower panel of Fig. 2a demonstrates that ${\rho }_{{xy}}^{\text{AHE}}$ (red line) is zero in SIO, which is expected because its ground-state is neither ferromagnetic nor ferrimagnetic, i.e., $M\sim 0$ (red line in Fig. 2b). As expected^10,11^, there exists a finite ${\rho }_{{xy}}^{\text{AHE}}$ in LSMO (red line top panel of Fig. 2a), but also in the case of the LSMO/SIO bilayers (see example in the lower panel of Fig. 2a and more data in Supplementary Fig. 1). Hereinafter, we define $\Delta {\rho }_{{xy}}^{\text{AHE}}$ as the value of ${\rho }_{{xy}}^{\text{AHE}}$ once ${M}_{z}$is saturated by ${H}_{z}$(green arrows in Fig. 2a). Note finally that the sign of the OHE in LSMO is opposite to that in SIO because carriers in manganites are holes while transport in SIO is dominated by the more mobile electrons^31^. Within a temperature range in which the magnetization is virtually constant (which is essentially the case here since $M\left(T\right)/M\left(0\right) \sim 1$ for *T* ≤ 100 K), the measured $\Delta {\rho }_{{xy}}^{\text{AHE}}$ changes with the temperature only through its relationship with the longitudinal resistivity ${\rho }_{{xx}}$, which as discussed above, is given by the inversion of the conductivity tensor and reads:^1^

1 $$\Delta {\rho }_{{xy}}^{\text{AHE}}\left(T\right)=\left|{\sigma }_{{xy}}^{\text{AHE}}\right|{\rho }_{{xx}}^{2}\left(T\right),$$

where ${\sigma }_{{xy}}^{\text{AHE}}$ is the anomalous Hall conductivity.

In Fig. 2b, we plot $\Delta {\rho }_{{xy}}^{\text{AHE}}\left(T\right)$ as a function of ${\rho }_{{xx}}^{2}\left(T\right)$ for a series of bilayers having similar LSMO thickness (${d}_{\text{L}}$ = 27.3 ± 6.3 nm) and varying SIO thickness (${d}_{\text{S}}$). The set of data points for each sample (symbol type) is obtained from measurements as those in Fig. 2a taken at various temperatures (20 K ≤ *T* ≤ 100 K). A linear scaling is observed for the whole range of ${d}_{\text{S}}$, including the sample without SIO (${d}_{\text{S}}$ = 0). This demonstrates that the anomalous Hall conductivity is independent of the scattering rate, and thus that it is an intrinsic contribution of the AHE driven by the topological properties of Bloch states in reciprocal space (see Supplementary Figs. 2 and 3 and Supplementary Note 1 for further supporting analysis). Note in Fig. 2b that the slope of $\Delta {\rho }_{{xy}}^{\text{AHE}}$ vs. ${\rho }_{{xx}}^{2}$, which following Eq. 1 corresponds to AHE conductivity ${\sigma }_{{xy}}^{\text{AHE}}$ of the bilayer system, decreases as the SIO thickness ${d}_{\text{S}}$ is increased. This is shown in more detail in Fig. 2c, which displays ${\sigma }_{{xy}}^{\text{AHE}}$as a function of ${d}_{\text{S}}$ for the full series of samples (circles). An abrupt decrease of ${\sigma }_{{xy}}^{\text{AHE}}$ is observed when ${d}_{\text{S}}$ increases, followed by saturation.

In order to understand the abrupt decrease of the AHE when a few layers of SIO are put in contact with LSMO, we analyze the AHE data using a circuit model that allows calculating the transverse ${\rho }_{{xy}}^{\text{AHE}}$ and longitudinal ${\rho }_{{xx}}$ resistivity of a multilayer based on the individual layers’. This model and the related algebra are described in Supplementary Fig. 4 and Supplementary Note 2. If we consider a relationship between the AHE and longitudinal resistivity of the form of Eq. 1, the model yields the following expression for the AHE conductivity:

2 $${\sigma }_{{xy}}^{\text{AHE}}=\frac{\Delta {\rho }_{{xy}}^{\text{AHE}}}{{\rho }_{{xx}}^{2}}=\frac{\mathop{\sum }\limits_{i=1}^{n}{d}_{i}{\sigma }_{{xy},i}^{\text{AHE}}}{\mathop{\sum }\limits_{i=1}^{n}{d}_{i}},$$

where $\Delta {\rho }_{{xy}}^{\text{AHE}}$ and ${\rho }_{{xx}}$ are the anomalous Hall and longitudinal resistivity expected in measurement on a heterostructure composed of *n* different materials, each of them having a thickness ${d}_{i}$, and anomalous Hall conductivity ${\sigma }_{{xy},i}^{\text{AHE}}=\Delta {\rho }_{{xy},i}^{\text{AHE}}/$ ${\rho }_{{xx},i}^{2}$. For the case investigated here, *n* = 2 (bilayers are made of LSMO and SIO as sketched in the left panel of Fig. 2d). If we assume that both materials (LSMO and SIO) keep their intrinsic ${\rm{AHE}}$ resistivity when combined in the heterostructures—that is, only LSMO shows a finite ${\sigma }_{{xy},\text{L}}^{\text{AHE}}$ while for SIO layer ${\sigma }_{{xy},\text{S}}^{\text{AHE}}$ = 0 as shown in Fig. 2a—then Eq. 2 yields:

3 $${\sigma }_{xy}^{\text{AHE}}=\frac{{d}_{\text{L}}}{(d_{\text{L}}+{d}_{\text{S}})}{\sigma }_{{xy},\text{L}}^{\text{AHE}},$$

where the AHE conductivity for LSMO ${\sigma }_{{xy},\text{L}}^{\text{AHE}}=\Delta {\rho }_{{xy},\text{L}}^{\text{AHE}}/{\rho }_{{xx},\text{L}}^{2}$ is known through the analysis shown in Fig. 2b. Thus we can readily calculate ${\sigma }_{{xy}}^{\text{AHE}}\left({d}_{\text{S}}\right.$) expected from Eq. 3, which is depicted in Fig. 2c (gray line). One can see that Eq. 3 gives a slow, gradual decay of ${\sigma }_{{xy}}^{\text{AHE}}$ with increasing ${d}_{\text{S}}$, which does not agree with the abrupt drop for small ${d}_{\text{S}}$—followed by near saturation—we observe in the experiments (solid circles in Fig. 2c). This shows that the assumption that LSMO and SIO keep their intrinsic AHE conductivity when combined in a heterostructure is not valid. In the following, we show that the abrupt drop in the AHE conductivity is explained by the emergence of AHE in SIO, whose sign is opposite to that in LSMO. We demonstrate below that the AHE originates in the proximity-induced magnetism in the narrow bands of the SIO.

### Magnetic characterization

**Fig. 3:**
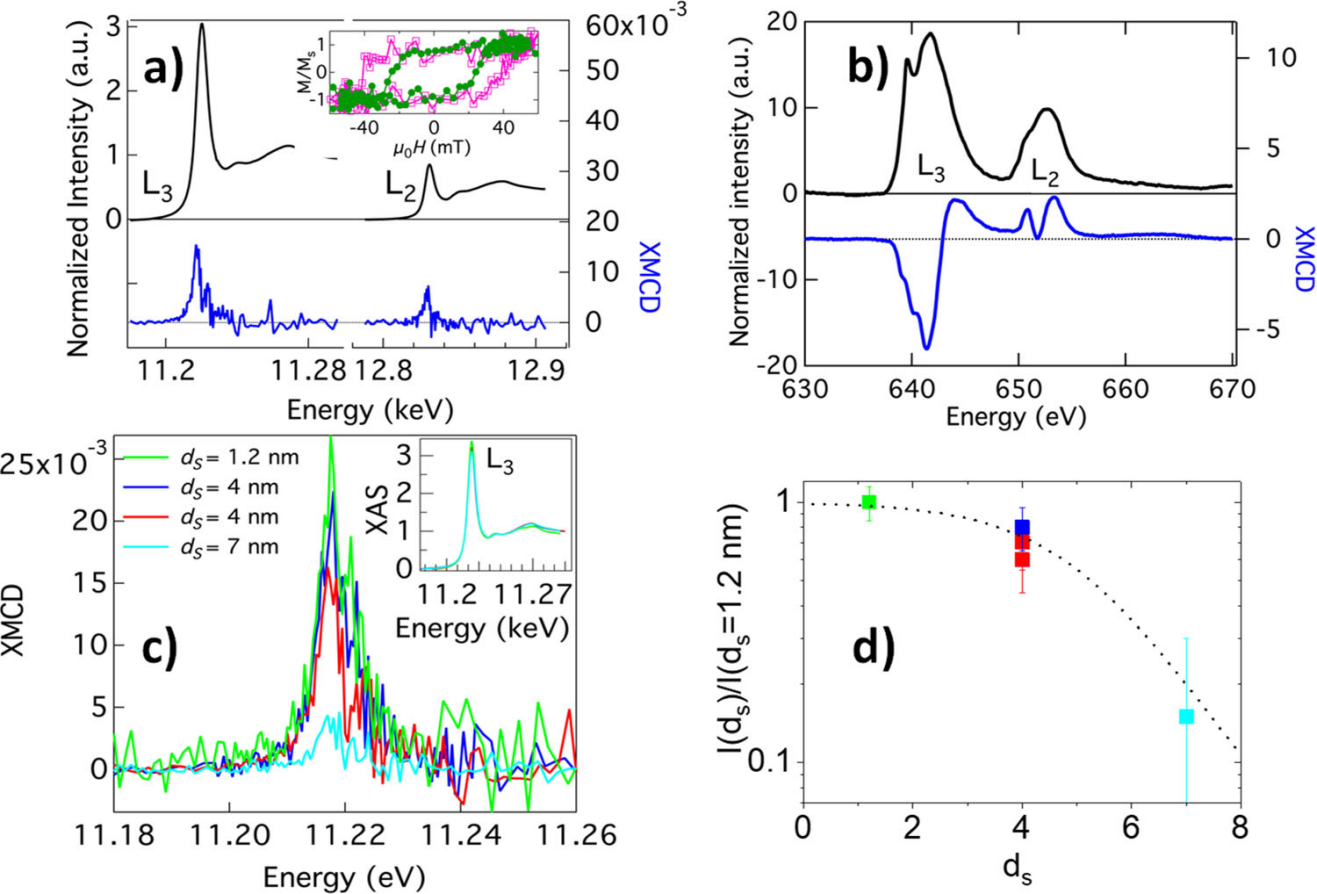
X-ray absorption spectroscopy. **a** Ir L_2,3_ XAS (black lines) and XMCD (blue lines) PFY spectra measured at *T* = 20 K under a saturating magnetic field of 55.6 mT applied in the [100] direction of a sample SrTiO_3_(100)//SrIrO_3_ (4 nm)/La_0.7_Sr_0.3_MnO_3_ (5 nm). Inset: L_3_ Ir hysteresis loop measured at *T* = 20 K and *E* = 11.217 keV with in-plane magnetic fields along with the [100] (magenta open squares) and [110] (green circles) directions, measured from the fluorescence and X-ray reflectivity intensities, respectively. **b** Mn XAS absorption spectra (black line) and XMCD (blue line) measured in the TEY mode at *T* = 50 K at remanence after saturating in a 280 mT magnetic field applied in the [110] direction. **c** XMCD intensities for samples STO//SIO(*d*_*S*_)/LSMO(5 nm) at the Ir L_3_ edge. For ${d}_{\text{S}}$ = 1.2 nm and 7 nm, *T* = 10 K and ${\mu }_{0}H$ = 60 mT. For ${d}_{\text{S}}$ = 4 nm, two nominally identical samples were measured, at various temperatures (20 K and 2 K) and magnetic fields (55.6 mT, 0.5 T). The intensity was independent of the magnetic field applied. The peak intensity at 2 K is higher than those at 20 K. Inset: Normalized XAS intensity. **d** The ratio between measured XMCD peak intensities of samples with larger SIO thicknesses and the peak intensity of the sample with the thinnest SIO ${d}_{\text{S}}$= 1.2 nm as a function of SIO thickness.

The magnetism in LSMO and SIO was studied by a combination of X-ray absorption spectroscopy (XAS) and X-ray magnetic circular dichroism (XMCD) at the Mn L_2,3_ and Ir L_2,3_ edges, conducted on samples SrTiO_3_(100)//SrIrO_3_ (${d}_{\text{S}}$)/La_0.7_Sr_0.3_MnO_3_ (5 nm), with ${d}_{\text{S}}$ = 1.2 nm, 4 nm, and 7 nm. The low thickness of the top LSMO layer was chosen to achieve good interface sensitivity. Magnetic fields were applied in-plane in either the [100] or [110] directions. Figures 3a and 3b show absorption spectra of a bilayer sample with ${d}_{\text{S}}$ = 4 nm for Ir (Fig. 3a) measured at *T* = 20 K, ${\mu }_{0}H$ = 55.6 mT and for Mn (Fig. 3b) at 50 K, ${\mu }_{0}H$ = 0 T. Measurements were conducted after field cooling in 280 mT in the case of Mn and, due to magnet limitations, in 55.6 mT in the case of Ir. The field of 55.6 mT was high enough to saturate magnetization in either field orientation (see below). XMCD measured in total electron yield (TEY) mode showed a robust magnetic moment of the Mn. XMCD spectra of the L-edge in partial fluorescence yield (PFY) mode showed clear evidence of Ir magnetic moment. Sum rules^32^ using L_2_ and L_3_ edge data at 20 K were used to obtain spin ($\left\langle {S}_{z}\right\rangle =0.003\pm 0.0007{\mu }_{\text{B}}$) and orbital (${\left\langle {L}_{z}\right\rangle =0.018\pm 0.001\mu }_{\text{B}}$) moments, which were aligned antiparallel to the Mn moment. Values of spin and orbital moments estimated from PFY using sum rules are considered a rough estimate due to sources of error such as the effect of the magnetic dipole moment, Tz, on the spin moment^32^ and self-absorption effects (expected to be limited due to the small thickness of the samples). Square-shaped hysteresis loops of the Ir moment (see inset to Fig. 3a) measured at *T* = 20 K and *E* = 11.217 keV had a coercivity of 40 mT and 24 mT, for *H* applied along [100] and [110] directions, respectively. A large ratio of the orbital to spin moment $\frac{{L}_{z}}{{S}_{z}}=6$ was found, much larger than the nominal $\frac{{L}_{z}}{{S}_{z}}=4$ characteristic of the SIO-214^25^, as previously reported for manganite iridate superlattices.

Induced magnetism in the iridate has been found previously in manganite/iridate superlattices with different interface reconstructions^25–27^ although, as discussed below, important differences result from our peculiar interface reconstruction. X-ray absorption spectroscopy experiments at the Ir L_3_ edge for samples with different SIO thicknesses are shown in Fig. 3c. XMCD spectra did not show any measurable shift (see inset to Fig. 3c) as thickness was changed indicating that the charge transfer mechanism observed in SrMnO_3_/SIO interfaces^33^ is inhibited here, probably by the two interfacial SrO rock salt blocks. The main panel of Fig. 3c shows XMCD spectra around the Ir L_3_ edge normalized to the XAS edge jump intensity for various samples. The XMCD peak intensity was maximum for the sample with ${d}_{\text{S}}$ = 1.2 nm and reduces with increasing ${d}_{\text{S}}$. For ${d}_{\text{S}}$ = 4 nm there is only a weak decrease of the XMCD signal while a strong suppression occurs for ${d}_{\text{S}}$ = 7 nm indicating that, since XMCD is normalized to the XAS signal, only part of the SIO layer has a magnetic moment. We can estimate the thickness of the interfacial SIO layer ${d}_{\text{I}}$ that has a magnetic moment by scaling the XMCD peak intensity to that measured for ${d}_{\text{S}}$ = 1.2 nm, as shown in Fig. 3d. The sigmoidal-like dotted line is a guide to the eye to show the exponential decrease of the intensity from which we estimate that ${d}_{\text{I}}$ = 2 nm. The Ir magnetic state can be discussed in connection to the atomic reconstruction of the interface uncovered by the STEM EELS experiment.

To address the origin of the induced ferromagnetism in the SIO layer separated from the LSMO by a double SrO layer, we have started by performing density functional theory (DFT) calculations on bulk SIO. We find that canted AFM ordering driven by the Dzyaloshinskii-Moriya interaction^22,34–36^ is stable when the local U on Ir d states is larger than ~1 eV (see Supplementary Fig. 5 and Supplementary Note 3). That is, the SIO is the neighborhood of a canted antiferromagnetic state triggered by enhanced electron correlation. Because of the small energy difference between different canted AFM states, less than ~10 meV/Ir, the weak superexchange interaction^37–42^ across the double SrO layers, evidenced by the antiferromagnetic alignment of the Mn and Ir moments, is enough to drive the observed magnetic state. It is worth pointing out that this superexchange interaction across double SrO layers has been observed in La_1.85_Sr_0.15_CuO_4_ (LSCO-214) cuprate/manganite interfaces^30^ where it also yields an antiferromagnetic (Cu–Mn) state.

### Discussion

The finding of induced magnetism in SIO at the interface with LSMO over a nanometric length scale suggests that the unexpected drop of the AHE conductivity (Fig. 2c) with increasing SIO thickness is produced by the emergence of AHE in SIO. Based on this idea, we analyzed the AHE data by modeling the heterostructures with a trilayer model including an interface layer as sketched in Fig. 2d. In essence, we consider that the SIO layer is divided into two sublayers: an interface layer “I” of thickness ${d}_{\text{I}}$ in which there exists induced magnetism and AHE, and a second layer “S0” of thickness ${d}_{\text{S}0}$ in which the intrinsic properties of SIO (in particular the absence of AHE) are preserved. We assume thus that there is a characteristic length scale ${d}_{\text{c}}$ over which the magnetic and AHE properties of SIO are affected by proximity with the LSMO. Consequently, in heterostructures in which the SIO thickness ${d}_{\text{S}}$ ≤ ${d}_{\text{c}}$, the entire SIO layer shows modified properties: ${d}_{\text{I}}$ = ${d}_{\text{S}}$. For heterostructures with ${d}_{\text{S}}$> ${d}_{\text{c}}$, ${d}_{\text{I}}$ = ${d}_{\text{c}}$ and ${d}_{\text{S}0}$ = ${d}_{\text{S}}-{d}_{\text{c}}$. To simplify the calculations, we assume that the magnetic and electronic properties are uniform within each of the three layers ${d}_{\text{L}}$, ${d}_{\text{I}}$ and ${d}_{\text{S}0}$.

We can now apply Eq. 2 to the three-layer system, using *n* = 3, with ${\sigma }_{{xy},\text{S}0}^{\text{AHE}}$= 0 since the intrinsic SIO properties are preserved within ${d}_{\text{S}0}$. Thus, Eq. 2 yields:

4 $${\sigma }_{{xy}}^{\text{AHE}}=\frac{{d}_{{\rm{L}}}{\sigma }_{{xy},\text{L}}^{\text{AHE}}+{d}_{{\rm{I}}}{\sigma }_{{xy},\text{I}}^{\text{AHE}}}{{d}_{{\rm{L}}}+{d}_{{\rm{S}}}},$$

Note that ${d}_{{\rm{L}}}$,${d}_{{\rm{S}}}$, ${\sigma }_{{xy}}^{{{\mathrm{AHE}}}}$, and ${\sigma }_{{xy},L}^{{{\mathrm{AHE}}}}$ and ${d}_{{\rm{c}}}$ (the upper limit of the interfacial layer thickness ${d}_{{\rm{I}}}$) can be estimated from the thickness of the layer with an induced magnetic moment at the SIO interface ${d}_{{\rm{c}}}=2$ nm. We can thus calculate the contribution to the anomalous Hall conductivity of the interfacial SIO layer with induced magnetism

5 $${\sigma }_{{xy},\text{I}}^{\text{AHE}}=\frac{\left({d}_{{\rm{L}}}+{d}_{{\rm{S}}}\right){\sigma }_{{xy}}^{\text{AHE}}-{d}_{{\rm{L}}}{\sigma }_{{xy},\text{L}}^{\text{AHE}}}{{d}_{{\rm{I}}}},$$

and plot it as a function of the SIO thickness ${d}_{{\rm{S}}}$ in Fig. 2e. One sees that our analysis in terms of the three-layers-model identifies an intrinsic contribution to the AHE conductivity, ${\sigma }_{{xy},\text{I}}^{\text{AHE}}$ which is negative and nearly independent of ${d}_{{\rm{S}}}$. In the following, we discuss the topological origin of this contribution and its connection to induced magnetism in the SIO layer.

It is important to remark that these results differ from the AHE measured recently in SrMnO_3_ (antiferromagnetic insulator) and SIO superlattices^26^. In that work the appearance of magnetic moment in Ir was accompanied by changes in the magnetism of the SMO layer (which becomes ferromagnetic); and (ii) the emergence of AHE in the heterostructures was ascribed to AHE in the SMO layer, not in the SIO one. Moreover, the AHE was positive, instead of the negative AHE in our work, and was found to be in the dirty metal limit instead of the intrinsic limit of our system. It is also worth pointing out that we did not observe the topological Hall effect THE theoretically predicted^43^ and experimentally observed^44^ in similar SIO/LSMO interfaces. THE results from topological spin textures (e.g., skyrmions), and shows a characteristic signature in the Hall resistivity as (antisymmetric) “humps” superposed to the AHE and OHA signal. However, we did not observe such a signature in our samples. This can be clearly recognized in the Hall data for an SIO/LSMO shown in Fig. 2a, as well as in the raw data displayed in Supplementary Fig. 1. The THE generally appears at the interface between ferromagnets with perpendicular magnetic anisotropy and spin-orbit materials and is driven by the interfacial Dzyaloshinskii-Moriya interaction (DMI). The absence of THE in our samples is likely connected to the interface reconstruction with double SrO planes, which weakens the interfacial DMI because longer Mn-Ir distance suppresses the effect of SOC. This argument is consistent with the fact that inserting an ultrathin insulating layer between SIO and LSMO also suppresses the THE as reported earlier^44,45^. The lack of THE, thus, rules out that the observed AHE results from non-collinear spins in the LSMO at the interface. Finally, notice that the strong negative contribution to the anomalous conductivity cannot result from a suppressed magnetization of the LSMO at the interface, which if any, would occur over a nanometric (1–2 nm) length scale producing only small changes in the anomalous Hall conductivity of the 25 nm thick LSMO layer. See Supplementary Figs. 6 and 7 and Supplementary Note 4.

The induced magnetism at the SIO interface provides an artificial realization of the necessary conditions for the AHE. Its independence on temperature (scattering rate) reflects its intrinsic character related to the topological properties of Bloch states, namely, the Berry curvature of the occupied states^3^. The Hall angle given by the ratio $\frac{{\sigma }_{{xy}}^{\text{AHE}}}{{\sigma }_{{xx}}}$ is typically small (well below 1%) in materials with trivial topology bands, since ${\sigma }_{{xy}}^{\text{AHE}}$ is small and ${\sigma }_{{xx}}$ is typically large. For the LSMO we find ${\sigma }_{{xy}}^{\text{AHE}}=6.54{(\Omega {{\mathrm{cm}}})}^{-1}$ yielding values of the anomalous Hall angle in the 0.1–0.2% (the range resulting from the temperature dependence of the longitudinal conductivity). LSMO can be regarded to have topologically trivial bands, although carriers have been proposed to acquire a real space Berry phase due to non-collinear spin structures mostly near *T*_C_^10,11^ and small values of the anomalous Hall conductivity are expected. On the other hand for SIO ${\sigma }_{{xy},I}^{\text{AHE}}=-63{(\Omega {{\mathrm{cm}}})}^{-1}$ is much larger, which considering ${\sigma }_{{xx}} \sim 500-1000{(\Omega {{\mathrm{cm}}})}^{-1}$ (see Supplementary Fig. 8 and Supplementary Note 5.) yields larger values of $\frac{{\sigma }_{{xy},I}^{\text{AHE}}}{{\sigma }_{{xx}}} \sim 6-13 \%$. These values are comparable, yet larger, than for SrRuO_3_^7^ or to MnSi^46,47^ or non-collinear antiferromagnet Mn_3_Sn^5^ or CoNb_3_S_6_^6^ with topologically non-trivial bands. Notably, this is despite the small values of the magnetic moment induced by the interfacial proximity interaction (${\sigma }_{{xy}}^{\text{AHE}}$ which is proportional to magnetization).

The large absolute value of ${\sigma }_{{xy},I}^{\text{AHE}}$ together with its independence on the scattering rate strongly suggests its intrinsic origin, i.e., the topological nature of the Bloch states in SIO. In fact, the topological band property in SIO has been the subject of great attention. Band structure calculations^48,49^ have shown band crossings forming a Dirac nodal ring in the U-R-X plane of the Brillouin zone. It was first proposed that its protection by the mirror symmetry of the *Pbnm* space group would render a topological semimetal. Later on, it was found that the Dirac degeneracy is lifted by epitaxial strain in thin films and that a small gap opens in thin films under epitaxial strain^22,50^. This showed that SrIrO_3_ has a Dirac line (ring) node that is protected by the *Pbmn* n-glide symmetry and is thus a non-symmorphic Dirac semimetal^51,52^. In contrast to such Dirac semimetallic behaviors which are more closely related to non-magnetic topological systems, our LSMO/SIO systems have proximity-induced magnetism. Thus, broken time-reversal, as well as inversion symmetries, should lift the Dirac degeneracy at nodal lines or points. However, such symmetry breakings could induce robust hot spots in the Berry curvature by lifting the Dirac degeneracy^53^. Furthermore, the stronger SOC in Ir than in Ru could lead to a much larger intrinsic AHE despite its weaker magnetism.

### Theoretical analysis

**Fig. 4:**
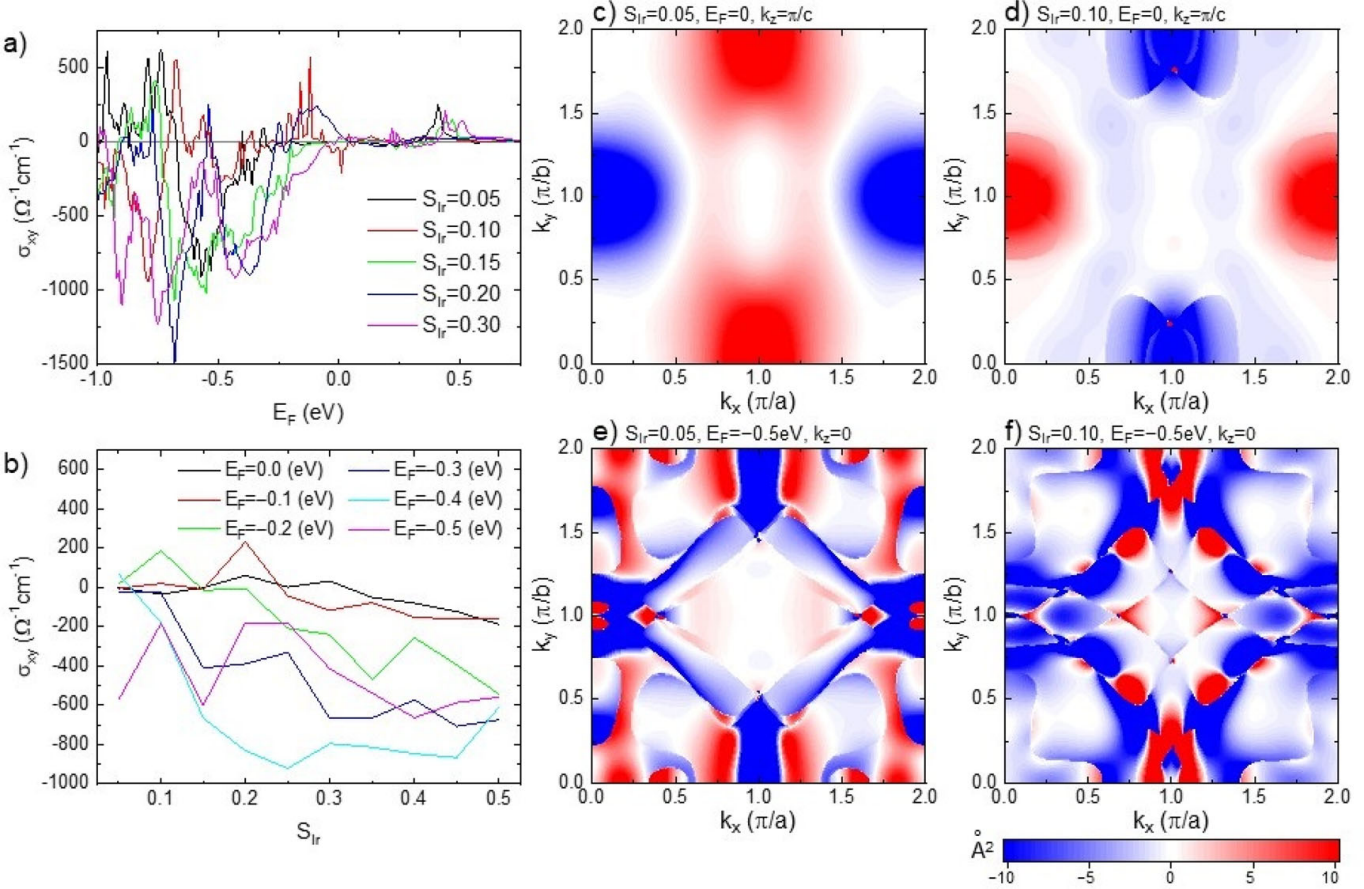
Theoretical analysis. **a** Intrinsic AH conductivity (**a**) as a function of the Fermi level ${E}_{F}$ with several values of spin polarization ${S}_{{\rm{Ir}}}=\left({n}_{{\rm{up}}}-{n}_{{\rm{down}}}\right)/2$ and (**b**) as a function of ${S}_{{\rm{Ir}}}$with several values of ${E}_{F}$. 2D plot of the Berry curvature for (**c**) ${S}_{{\rm{Ir}}}=0.05$ at ${E}_{F}=0$ and ${k}_{z}=\pi /c$, (**d**) ${S}_{{\rm{Ir}}}=0.10$ at ${E}_{F}=0$ and ${k}_{z}=\pi /c$, (**e**) ${S}_{{\rm{Ir}}}=0.05$ at ${E}_{F}=-0.5$ eV and ${k}_{z}=0$, and (**f**) ${S}_{{\rm{Ir}}}=0.10$ at ${E}_{F}=-0.5$ eV and ${k}_{z}=0$. ${n}_{{\rm{up}}\left({\rm{down}}\right)}$ is the spin-up (down) electron density per Ir site.

In order to gain insights into the topological origin of the intrinsic AHE, we carried out theoretical analyses based on density functional theory calculations. To minimize the complexity arising from the interface with LSMO, we considered bulk SIO with the appropriate lattice constants as detailed in the “Methods” section. The proximity coupling with LSMO is simulated by performing constrained magnetic moment calculations. Here, the direction of Ir spin polarization ${S}_{{\rm{Ir}}}$ is fixed along the *z* direction and their magnitude is varied. We then compute the AH conductivity ${\sigma }_{{xy}}$ of SIO due to “proximity-induced” magnetism. The resultant ${\sigma }_{{xy}}$ is summarized in Fig. 4a as a function of the Fermi level ${E}_{F}$ with several values of Ir spin polarization ${S}_{{\rm{Ir}}}$ and in Fig. 4b as a function of ${S}_{{\rm{Ir}}}$ with several values of ${E}_{F}$. Since by definition spin polarization is oppositely directed to magnetic moment, the positive SIO spin polarization corresponds to the experimental situation of Ir moments anti-aligned to Mn moments at the interface. The negative sign of ${\sigma }_{{xy}}$ obtained in the calculations is thus consistent with the experiment. To our surprise, ${\sigma }_{{xy}}$ reaches a very large value $-1000{(\Omega {cm})}^{-1}$ at certain condition. The non-monotonic behavior of ${\sigma }_{{xy}}$ as a function of ${E}_{F}$ or ${S}_{{\rm{Ir}}}$, including the sign change, resembles that of SrRuO_3_ as reported by Fang et al.^7^, suggesting the same origin, i.e., magnetic monopoles in momentum space. To check this scenario, we examined the Berry curvature with different parameter sets; examples are shown in Fig. 4c–f. One potential origin of the large ${\sigma }_{{xy}}$ is a Dirac nodal line in the U-R-X plane in the momentum space as predicted by Zeb et al. and Carter et al.^47,48^. This could also induce magnetic monopoles and contribute to ${\sigma }_{{xy}}$ when the Fermi level ${E}_{F}$ is close to 0, corresponding to stoichiometric SIO. At ${E}_{F}=0$ with ${S}_{{\rm{Ir}}}=0.05$, this induces only a moderate enhancement in the Berry curvature at $({k}_{x},{k}_{y},{k}_{z}) \sim \left(\frac{\pi }{a},0,\frac{\pi }{c}\right)$ and $\left(\frac{\pi }{a},2\frac{\pi }{b},\frac{\pi }{c}\right)$ [see Fig. 4c] because the line node is away from ${E}_{F}=0$. A slight enhancement in ${S}_{{\rm{Ir}}}$ is found to shift the line node closer to ${E}_{F}=0$ and induce very sharp peaks in the Berry curvature at $({k}_{x},{k}_{y},{k}_{z}) \sim \left(\frac{\pi }{a},0.25\frac{\pi }{b},\frac{\pi }{c}\right)$ and $\left(\frac{\pi }{a},1.75\frac{\pi }{b},\frac{\pi }{c}\right)$,see Fig. 4d. With some values of ${S}_{{\rm{Ir}}}$ with ${E}_{F}=0$, the theoretical ${\sigma }_{{xy}}$ could become comparable to the experimental estimation for SIO, $\sim -40{\Omega }^{-1}{{\rm{cm}}}^{-1}$ (see Figs. 4a and 4b). At negative ${E}_{F}$, corresponding to hole doping, a further enhancement appears, ${\sigma }_{{xy}} \sim -1000{\Omega }^{-1}{{\rm{cm}}}^{-1}$, as shown in Figs. 4a and 4b. This is ascribed to magnetic monopoles induced at avoided band crossings in the presence of the SOC and magnetic ordering. As shown in Figs. 4e and 4f the Berry curvature consists of multiple peaks with different magnitude and strongly supports this scenario.

To gain insight into which momentum and energy regime contributes to the large ${\sigma }_{{xy}}$, a Dirac nodal line near the U point and the stoichiometric Fermi level or avoided band crossing in hole-doped regime or both, we have analyzed band dispersion near the Fermi level and Berry curvature. Supplementary Fig. 9 shows plots of the dispersion relations near the Fermi level, 2D Fermi surfaces, and the separation between two adjacent bands, as well as Berry curvature for different cases. Nodal lines existing in non-magnetic SrIrO_3_ are unstable with respect to the magnetism, i.e., nonzero magnetic moment eliminates the nodal line and opens a gap. As the magnetic moment increases, different bands move up or down, creating many different kinds of band crossings. The plots show that such band crossing indeed produces the enhancement in the Berry curvature (See Supplementary Note 6). The semi-quantitative agreement in ${\sigma }_{{xy}}$ between our experimental estimation and theoretical calculations strongly suggests that the AHE in SIO-LSMO heterostructures is of topological origin, arising from magnetic monopoles in momentum space. Further analysis of the precise valence of Ir ions and induced magnetic moment may ultimately clarify the subtle difference in the location of magnetic monopoles.

A final remark is in order concerning the relative importance of the “side jump” (extrinsic) mechanism which is also independent of the scattering rate and can thus not be inferred from the relationship between the transverse and longitudinal resistivity deduced from transport measurements. As shown previously, the side-jump contribution is smaller than the intrinsic contribution in a factor of E_SO_/E_F_ which makes the intrinsic contribution dominant over a wide range of the scattering strength in clean or moderately dirty cases^4^.

In summary, the emerging intrinsic AHE observed in bilayers La_0.7_Sr_0.3_MnO_3_/SrIrO_3_ uncovers an exciting scenario of the interplay between topology and correlations at the 3*d*/5*d* interface. The symmetry breaking by the proximity-induced magnetism at the interface conspires with the strong spin-orbit interaction of the iridate to yield the hot spots of the integrated Berry curvature at nodal lines and band anticrossings, providing an artificial realization of the minimal model of the AHE^3^. It is very important that, despite the small values of the magnetic moment induced in the iridate by proximity to the manganite, the amplification by the topological properties of the iridate is so large that the anomalous Hall angle reaches values among the largest reported in the literature. The theoretical results show that the topological amplification of the AHE we have uncovered can be made even larger by hole doping, opening the door to the external tuning topological properties, an intriguing new direction towards future topological spintronics and spin-orbitronics at correlated oxide interfaces^54^ with strong spin-orbit interaction.

## Methods

### Sample growth

Samples were grown using a high-pressure sputtering system in a pure oxygen (2.8 mbar) atmosphere from stoichiometric La_0.7_Sr_0.3_MnO_3_ (LSMO) and SrIrO_3_ (SIO) targets. The growth temperature was 650 °C for SIO and 900 °C for LSMO. The high-pressure growth allows for a high degree of thermalization of extracted species before they arrive in the substrate. This technique has shown epitaxial growth of oxides with good epitaxial properties^37,39^.

### STEM EELS

Aberration-corrected scanning transmission electron microscopy (STEM) and electron energy loss spectroscopy (EELS) measurements have been obtained using a JEOL JEM ARM200cF operated at 200 kV using a condenser lens aperture of 1 mm. The specimens were prepared by mechanical grinding and polishing and Ar ion milling at grazing incidence in a Fischione 1010 ion mill. To get information about detailed plane sequence cross-section samples were prepared in [100] and [110] zone axes. EELS composition line scans were measured at element-specific absorption edges (Mn L_2,3_, La M_4,5_, and Ir M_4,5_, Sr L_2,3_) using a Gatan Quantum EEL spectrometer in dual EELS mode. EELS intensities at element-specific lines were obtained from multiple linear least-square (MLLS) methods.

### Transport measurements

The longitudinal and transverse dc resistances, ${R}_{{xx}}$ and ${R}_{{xy}}$, were measured as a function of the magnetic field H (applied perpendicular to the film plane) for different temperatures in the range 5–300 K using a closed-cycle refrigerator equipped with a superconducting magnet. The resistance is defined as ${R}_{{xx}}={V}_{{xx}}/{I}_{{xx}}$ and ${R}_{{xy}}={V}_{{xy}}/{I}_{{xx}}$, with ${I}_{{xx}}$ the injected dc current and ${V}_{{xx}}$ and ${V}_{{xy}}$ the voltages measured parallel and perpendicular to the injected current. Voltage offsets were removed by inverting the current sign and averaging the measured voltage. The pure Hall signal, which is “odd” with respect to the applied field, was calculated as $\langle {R}_{{xy}}\left(H\right)\rangle =({R}_{{xy}}\left(+H\right)-{R}_{{xy}}\left(-H\right))/2$. This allows removing any spurious contribution to ${R}_{{xy}}$ resulting from the electrodes’ misalignment and the strong magnetoresistance of LSMO (which is “even” with respect to the applied field). We calculated the resistivity ${\rho }_{{xx}}$ and ${\rho }_{{xy}}$ via the Van der Pauw method from the resistances obtained as described above, by considering the films’ dimensions and thickness.

### X-ray measurements

X-ray absorption spectroscopy (XAS) and X-ray magnetic circular dichroism (XMCD) measurement were conducted at the Advanced Photon Source (Argonne National Laboratory). The Mn L_2,3_ edge was probed at the APS beamline 4-ID-C (soft X-rays) and the total electron yield (TEY) and reflectivity signals were measured. The Ir L_2,3_ edge was probed at the APS beamline 4-ID-D (hard X-rays), where the partial fluorescence yield and reflectivity signals were measured.

### DFT calculations

First-principles DFT calculations were performed using the generalized gradient approximation^55^ and the projector augmented wave (PAW) pseudopotentials^56^ as implemented in the Vienna ab initio Simulation Package (VASP) code^57,58^. To better describe the strong correlation effect, the local Hubbard repulsion^59^ with *U* = 2 eV is included for Ir d states^60^. The spin-orbit coupling (SOC) is also turned on. To minimize the complexity arising from the interface with LSMO, we considered bulk SIO with the experimental lattice constants, $a=5.5617\mathring{\rm{A}}$, $a=5.5909\mathring{\rm{A}}$, and $c=7.8821\mathring{\rm{A}}$ with corrections^61,62^. The proximity coupling with LSMO is simulated by performing constrained magnetic moment calculations. Here, the direction of Ir moments is fixed along the crystallographic *z* direction and their size is varied.

Following a self-consistent calculation with total energy convergence of ${10}^{-5}$ eV, the maximally localized Wannier functions^63^ were constructed using the WANNIE90 code^64^ from the *ab* initio ground-state wave function. In the disentanglement process, 112 Wannier functions were chosen as initial projections including the *d* orbitals of Ir atoms and the *p* orbitals of O atoms. Finally, the AHC was calculated by computing the Berry curvature using the Wannier interpolation approach which is also implemented in the WANNIER90 code^64,65^.

## Supplementary information

Supplementary Information

## Data Availability

Data are available upon request from the authors.
