# Supplementary material for: Large intrinsic anomalous Hall effect in SrIrO3 induced by magnetic proximity effect
Source: Nat Commun. 2021 Jun 2;12:3283. doi: 10.1038/s41467-021-23489-y (PMC8172877; doi:10.1038/s41467-021-23489-y)
Supplement: Supplementary file 1 — Supplementary Information [file 41467_2021_23489_MOESM1_ESM.pdf]

# Supplementary Information

## Supplementary Figure 1

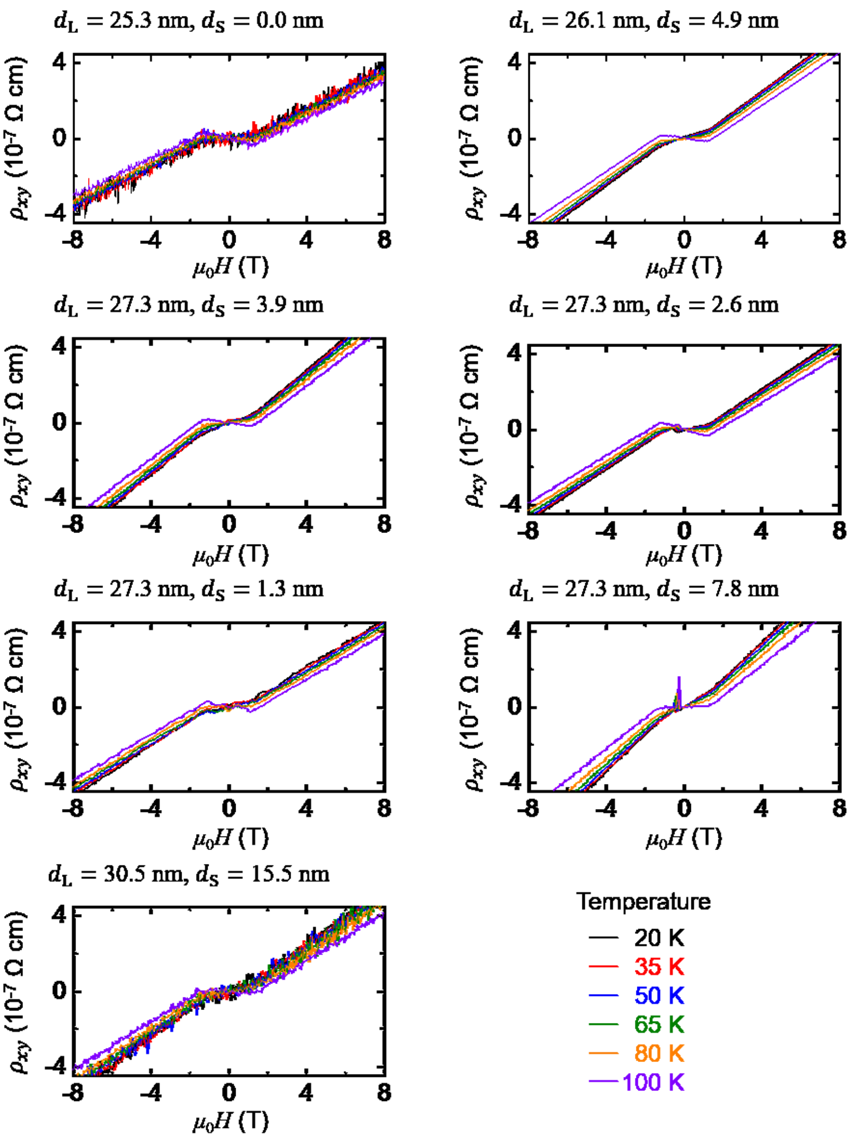


**Supplementary Figure 1** Hall resistivity for a series of samples with LSMO and SIO thickness indicated in the label. The color code indicates the temperature at which the measurement was carried.

## Supplementary Figure 2

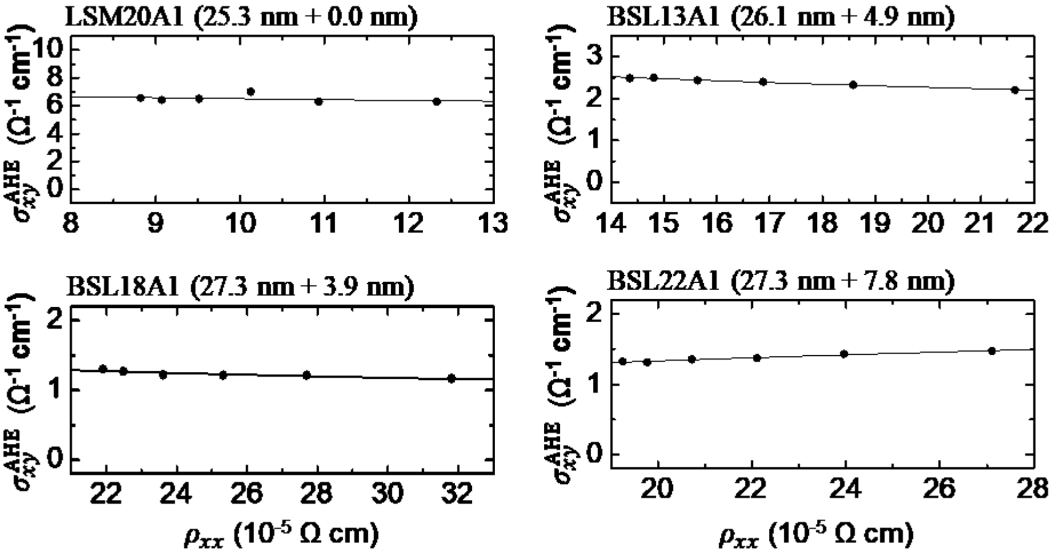


**Supplementary Figure 2** Plots of the anomalous Hall conductivity of $\sigma_{xy}^{\mathrm{AHE}}(T)$ as a function of $\rho_{xx}(T)$ for various heterostructures

## Supplementary Figure 3

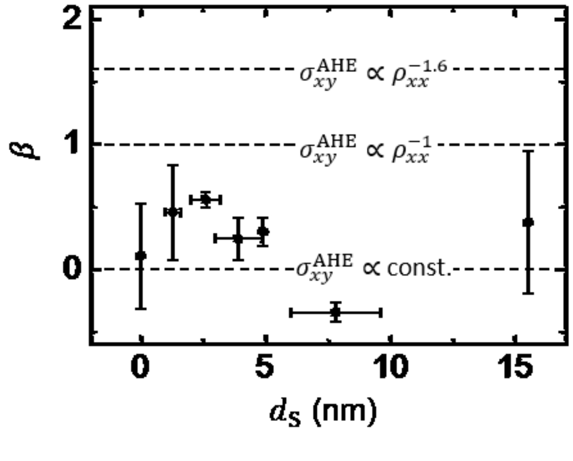


**Supplementary Figure 3**: Plots of the (longitudinal) resistivity exponent $\beta$ for all studied samples as a function of the SIO thickness, $d_S$.

# Supplementary Figure 4

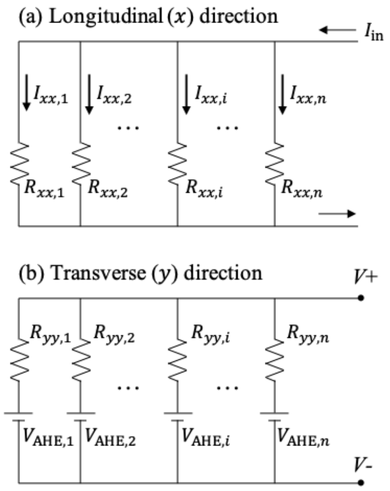


**Supplementary Figure 2** Circuit diagram along (a) longitudinal and (b) transverse directions for AHE in $n$-multilayers. $I_{xx,i}$ indicates current flows of each layer. $R_{xx,i}$ and $R_{yy,i}$ are the longitudinal resistivity in the $x$ and $y$ directions. $V_{\mathrm{AHE},i}$ is the AHE voltage induced by $I_{xx,i}$ in each layer $i$.

# Supplementary Figure 5

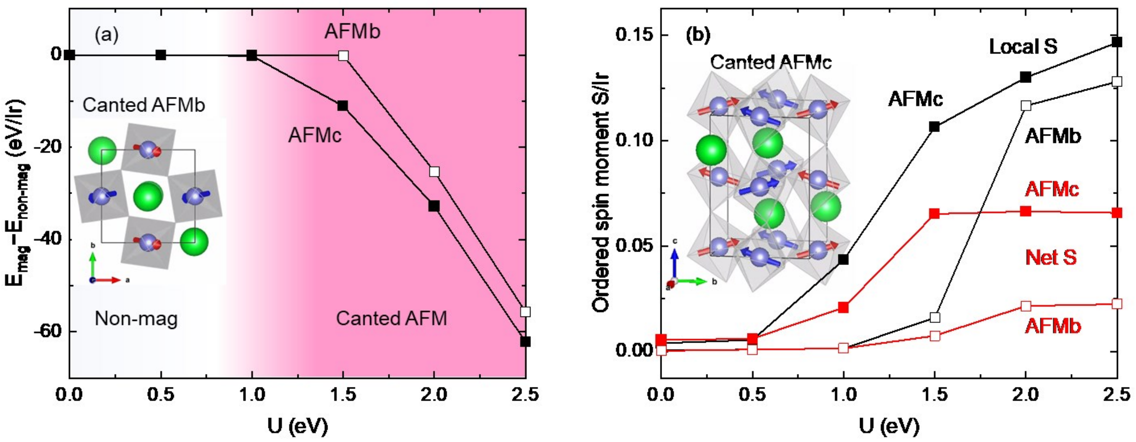


**Supplementary Figure 5** (a) Energy difference between magnetic states and a non-magnetic state. This quantity becomes negative at U>~1ev, indicating magnetic transition. (b) Ordered spin moment S/Ir (local value shown in black and net value shown in red). The inset of (a) shows the spin configuration of canted AFMb (the net moment along the b direction), and the inset of (b) the spin configuration of canted AFMc (the net moment along the c direction), The inset of (a) shows the spin configuration of canted AFMb (the net moment along the b direction), and the inset of (b) the spin configuration of canted AFMc (the net moment along the c direction). These figures are generated using VESTA [1].

## Supplementary Figure 6

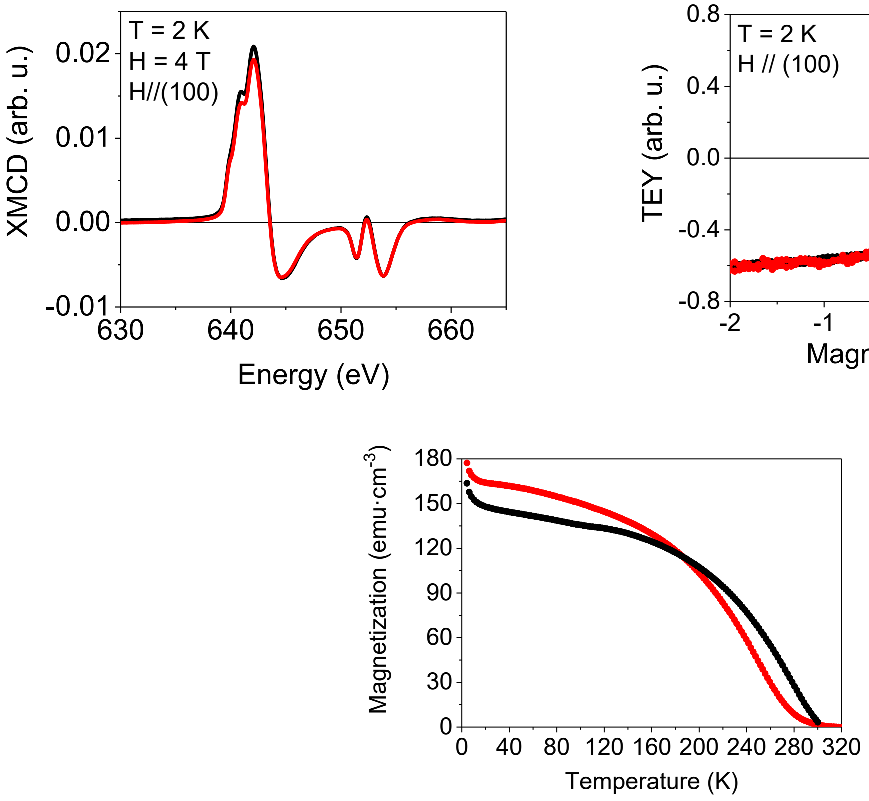


**Supplementary Figure 6**: XMCD signal at the Mn L2,3 edges at T = 2 K with an applied magnetic field $\mu_0 H$ =4 T (left). TEY XMCD hysteresis loop with magnetic field applied in the [100] direction. Samples are SIO (4.4 nm)/LSMO (7 nm) (black symbols) and SIO (4.4 nm)/BTO (2.4 nm)/LSMO (7 nm) (red symbols). Lower panel. SQUID magnetization vs temperature after field cooling in 1000 Oe field for the same two samples.

## Supplementary Figure 7

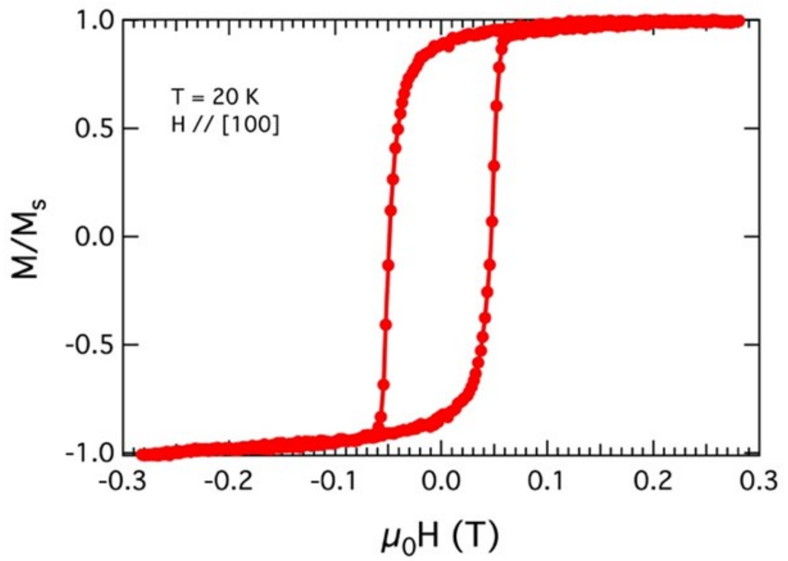


**Supplementary Figure 7**. XMCD hysteresis loops at the Mn $L_3$ edge of a STO/SIO (4m)/LSMO (5nm) bilayer measured with magnetic field aligned with the in plane [100] direction.

## Supplementary Figure 8

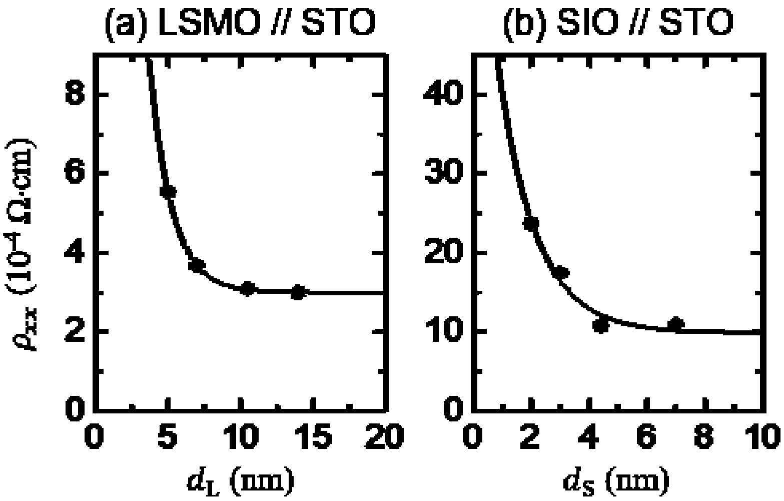


**Supplementary Figure 8.** Thickness dependence of longitudinal resistivity at $T = 100$ K in (a) $La_{0.7}Sr_{0.3}MnO_3(d_L)$//$SrTiO_3$ and (b) $SrIrO_3(d_S)$//$SrTiO_3$. Lines are guides to the eye.

## Supplementary Figure 9

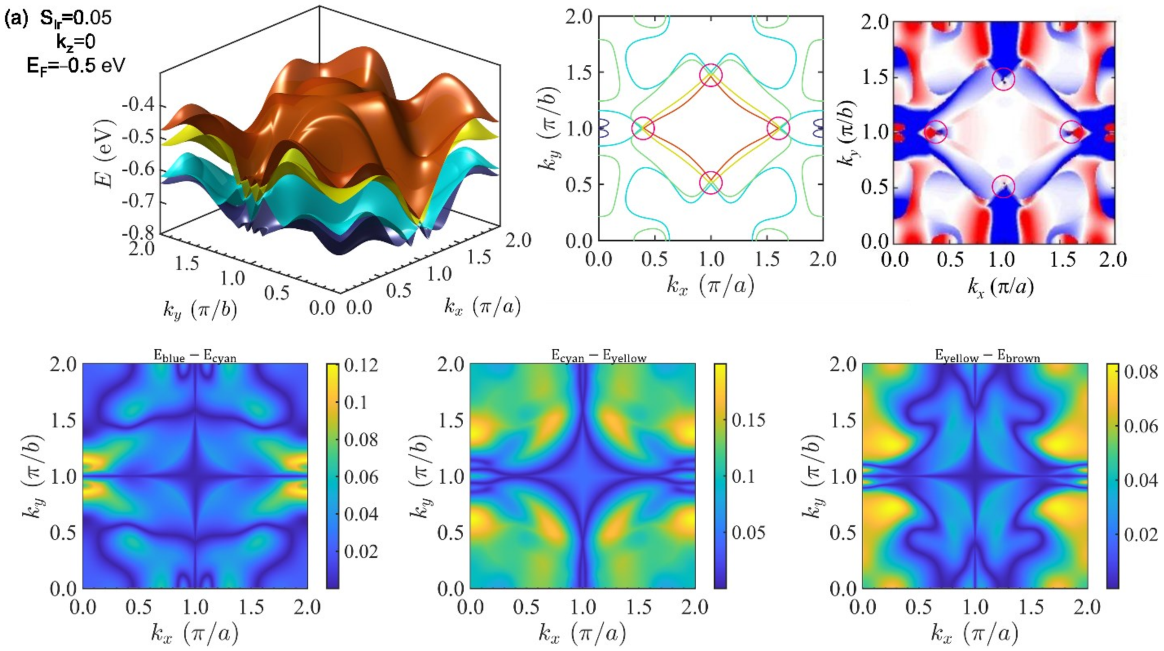


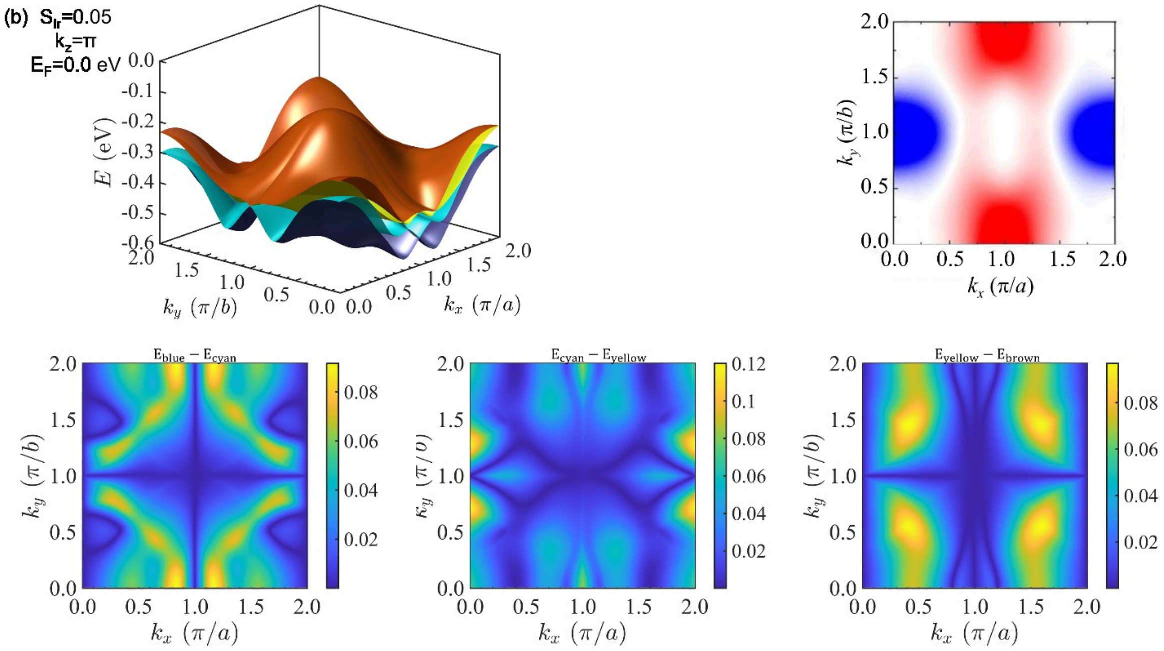

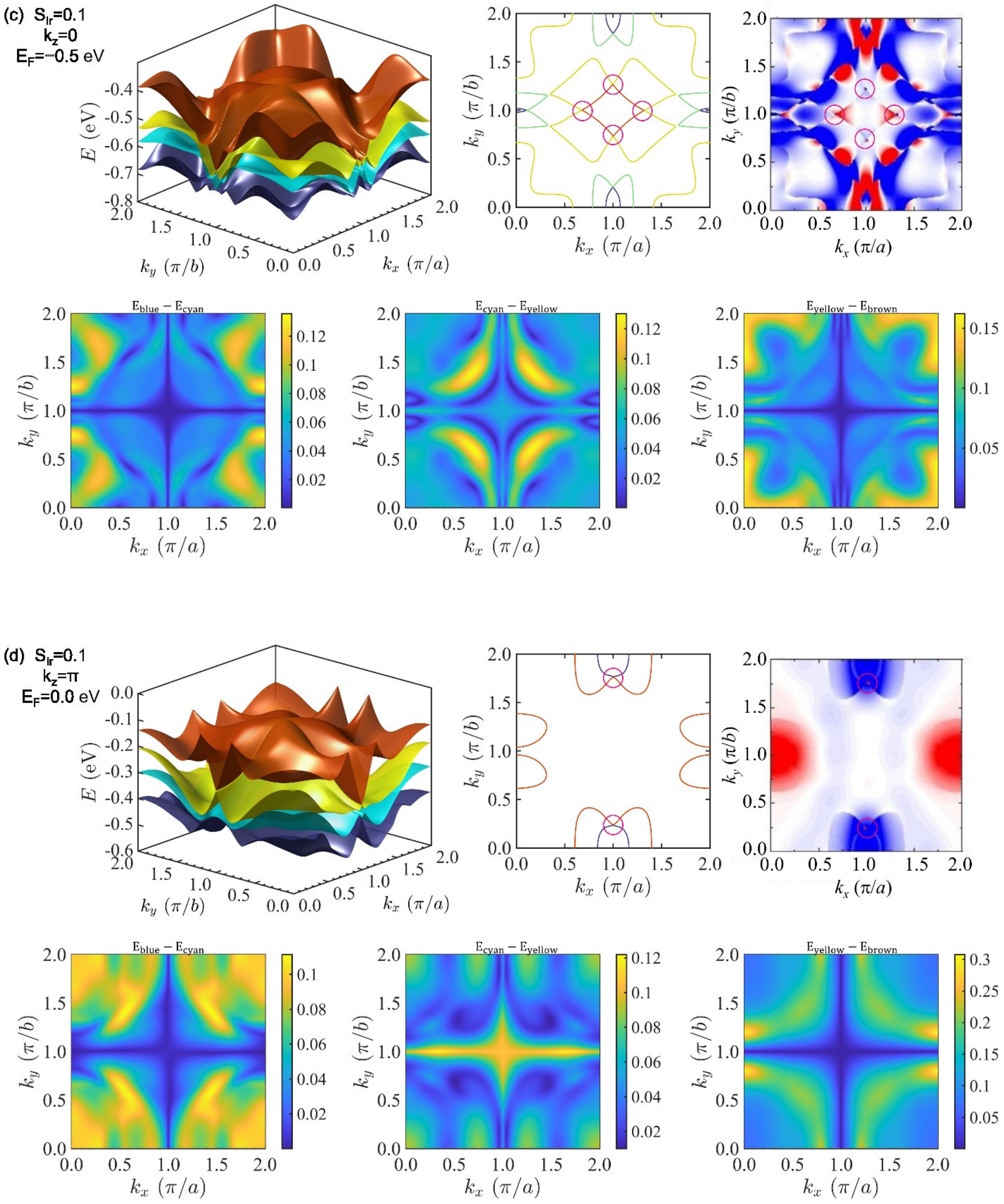


**Supplementary Figure 9.** Summary of density functional theory results for $S_{Ir}$=0.05 (a,b) and $S_{Ir}$=0.1 (c,d). On each panel, the top left figure shows the band dispersion near the Fermi level ($E_F$), the top middle two-dimensional Fermi surface, the top right two-dimensional Berry curvature as functions of ($k_x$,$k_y$). The bottom three figure show the energy separation between two neighboring bands as a function of ($k_x$, $k_y$). In (a) and (c) momentum along the third direction is taken as $k_z$=0 with $E_F$=-0.5 eV, and in (b) and (d) $k_z$=p with $E_F$ =0 (not shifted). (b) does not have the top middle panel because $E_F$ is inside the gap.

**Supplementary note 1. Relationship between $\sigma_{xy}^{\mathrm{AHE}}$ and $\rho_{xx}^2$**

Figure 2b in the main text implies that the AHE resistivity is roughly proportional to $\rho_{xx}^2$ (or, equivalently, that $\sigma_{xy}^{\mathrm{AHE}}$ in nearly independent of the longitudinal resistivity, $\rho_{xx}$. An alternative way to illustrate that conclusion is as follows.

By definition, we can obtain the AHE conductivity from the inversion of the conductivity tensor $\sigma_{xy}^{\mathrm{AHE}}(T) = \rho_{xy}^{\mathrm{AHE}}(T)/\rho_{xx}^2(T)$. In Supplementary Figure 3, we plot $\sigma_{xy}^{\mathrm{AHE}}(T)$ as a function of $\rho_{xx}(T)$ for various heterostructures.

One can see that, in all cases, $\sigma_{xy}^{\mathrm{AHE}}(T)$ is very weakly dependent on $\rho_{xx}(T)$. Thus, if one considers the relationship $\sigma_{xy}^{\mathrm{AHE}}(T) \propto \rho_{xx}^{-\beta}(T)$, one finds that for our experimental data the exponent $\beta$ is around $\sim 0$ (consistent with intrinsic AHE), and clearly further away from $\beta = 1.6$ and $\beta = 1$ expected in other scenario (extrinsic AHE) [4]. That is illustrated in Supplementary Figure 4, which gathers the exponent $\beta$ for all studied samples as a function of the SIO thickness $d_S$:

**Supplementary note 2. Resistor model of the electrical conduction in the heterostructure.**

The analysis of the anomalous Hall effect (AHE) in a multilayer system is based on the model developed in previous studies [2, 3] for the AHE in metallic multilayers.

The in-parallel conduction across the different layers ($i = 1, 2, \cdots, n$) leads to a distribution of the longitudinal (along $x$ ) current $I_{\mathrm{in}}$ injected in the multilayer, each layer "$i$" carrying

$I_{xx,i}$ according to its longitudinal resistance $R_{xx,i} = \frac{\rho_{xx,i}}{d_i}(\frac{l_x}{w_x})$, (Supplementary Figure 2 (a)). $\rho_{xx,i}$ and $d_i$ are the longitudinal resistivity and thickness of each layer. $l_x$ and $w_x$ are the length and width of the sample along the $x$ direction, which is the same for all layers.

To obtain the analytic form of the AHE conductivity $\sigma_{xy}^{\mathrm{AHE}} = \Delta\rho_{xy}^{\mathrm{AHE}}/\rho_{xx}^2$, we need to calculate the total longitudinal resistivity, $\rho_{xx}$, and anomalous Hall resistivity, $\Delta\rho_{xy}^{\mathrm{AHE}}$. $\rho_{xx}$ is calculated by considering the in-parallel conduction across the different layers as in the equivalent circuit of Supplementary Figure 2 (a):

$$\rho_{xx} = \left(\sum_{i=1}^{n} d_i\right)\left\{1/\left(\sum_{i=1}^{n}\frac{1}{\rho_{xx,i}/d_i}\right)\right\} = \eta\sum_{i=1}^{n} d_i, \qquad [1]$$

where $n$ is the total number of layers of the heterostructure, and $\eta = 1/\left(\sum_{i=1}^{n}\frac{1}{\rho_{xx,i}/d_i}\right)$.

The longitudinal current ($I_{xx,i}$) across each layer,

$$I_{xx,i} = \frac{I_{\mathrm{in}}}{\rho_{xx,i}/d_i}\left\{1/\left(\sum_{i=1}^{n}\frac{1}{\rho_{xx,i}/d_i}\right)\right\} = I_{\mathrm{in}}\frac{\eta}{\rho_{xx,i}/d_i} \quad , \qquad [2]$$

induces an AHE voltage in each of them,

$$V_{\mathrm{AHE},i} = I_{xx,i}\frac{\Delta\rho_{xy,i}^{\mathrm{AHE}}}{d_i}, \qquad [3]$$

which is different in each layer due to the different Hall resistivity $\Delta\rho_{xy,i}^{\mathrm{AHE}}$ and current $I_{xx,i}$. layer "$i$" . However, due to the boundary conditions, the total voltage along the transverse direction (along y), which is what is measured in the experiments and we denote $V_{\mathrm{AHE}}$, must

be the same within all the layers. This requires current flow in the transverse direction (along y), $I_{yy,i}$ , which generates (in each layer) a transverse voltage that compensates the difference between the individual Hall voltages $V_{\mathrm{AHE},i}$ and $V_{\mathrm{AHE}}$. The equivalent circuit is shown in Fig. S2 (b). The current $I_{yy,i}$ may have different sign in each of layers, depending on whether $V_{\mathrm{AHE},i} < V_{\mathrm{AHE}}$ or $V_{\mathrm{AHE},i} > V_{\mathrm{AHE}}$ , so that the total current along the y direction is zero, $\sum_i I_{yy,i} = 0$. From the above, and considering Supplementary Equations 2 and 3, we obtain $V_{\mathrm{AHE}}$, as well as the AHE resistivity, $\Delta\rho_{xy}^{\mathrm{AHE}}$,

$$V_{\mathrm{AHE}} = \left(\sum_{i=1}^{n} \frac{V_{\mathrm{AHE},i}}{\rho_{yy,i}/d_i}\right)\left\{1/\left(\sum_{i=1}^{n} \frac{1}{\rho_{yy,i}/d_i}\right)\right\} = I_{\mathrm{in}} \sum_{i=1}^{n} \frac{\Delta\rho_{\mathrm{AHE},i}}{d_i}\left[\frac{\eta}{\rho_{xx,i}/d_i}\right]^2 \quad [4]$$

$$\Delta\rho_{xy}^{\mathrm{AHE}} = \left(\sum_{i=1}^{n} d_i\right)\frac{V_{\mathrm{AHE}}}{I_{\mathrm{in}}} = \eta^2 \left(\sum_{i=1}^{n} d_i\right)\sum_{i=1}^{n}\left[d_i \frac{\Delta\rho_{\mathrm{AHE},i}}{{\rho_{xx,i}}^2}\right]. \quad [5]$$

Here we assume that the longitudinal resistivity along in the $x$ and $y$ directions are identical, i.e., $\rho_{xx,i} = \rho_{yy,i}$. Note that, for $n$ = 2, Supplementary Equation 5 is corresponds to the case studied in [2, 3]. Finally, from Supplementary Equations 1 and 5, the AHE conductivity, $\sigma_{xy}^{\mathrm{AHE}}$, is obtained

$$\sigma_{xy}^{\mathrm{AHE}} = \frac{\Delta\rho_{xy}^{\mathrm{AHE}}}{\rho_{xx}^2} = \left(\sum_{i=1}^{n} d_i\right)^{-1} \sum_{i=1}^{n}\left[d_i \frac{\Delta\rho_{xy,i}^{\mathrm{AHE}}}{{\rho_{xx,i}}^2}\right] = \left(\sum_{i=1}^{n} d_i\right)^{-1} \sum_{i=1}^{n} d_i\, \sigma_{xy,i}^{\mathrm{AHE}} \quad [6]$$

which corresponds to Eq. 2 in the main text.

**Supplementary note 3. Origin of induced ferromagnetism in SIO.**

To address the origin of the induced ferromagnetism in the SIO layer separated from the LSMO by a double SrO layer, we carry out additional density functional theory (DFT) calculations with +U collections on Ir d states considering various magnetic states. We find that canted AFM ordering is stable when the local U on Ir d states is larger than ~1eV (see Supplementary Figure 5,). The most stable magnetic state is found to be a canted AFM with the net Ir spin moment pointing along the c direction, for short AFMc. Another canted AFM with the net Ir spin moment pointing along the b direction, i.e., AFMb, is also found at slightly higher in energy ~10meV/Ir than the AFMc. That is, the SIO is the neighborhood of a canted antiferromagnetic state triggered by enhanced electron correlation at low thickness. Because of the small energy difference between different AFM states, the weak superexchange interaction across the interfacial double SrO layers is enough to stabilize the observed magnetic state, with Mn and Ir moments aligned antiferromagnetically.

**Supplementary note 4. X-ray absorption analysis of the Mn interface magnetization**

In this section we show evidence from XMCD and magnetization experiments that there is not significant suppression of the LSMO magnetization at the manganite / iridate interface.

We have prepared a bilayer STO(100)//SIO(4.4nm)/LSMO(7nm)) and a bilayer with the same thickness of LSMO and SIO but with a BaTiO3 (BTO) spacer in between the two layers (STO(100)//SIO(4.4nm)/BTO (2.4nm)/LSMO(7nm)). The insulating BTO layer will avoid any possible depression of the LSMO magnetization in contact with the SIO and also will suppress

any possible magnetic interaction between LSMO and SIO layers across the interface.

We measured XAS and XMCD signal at the Mn $L_{2,3}$ edge (See Supplementary Figure 6) . The XAS signal was measured in grazing incidence mode, and the magnetic field was applied in (100) direction of the sample.  Figure R1 shows XMCD spectra of the two samples at a temperature T = 2 K. The applied magnetic field was high enough (H = 4 T), to ensure that the magnetization is saturated in all samples. Both samples had comparable intensities of the XMCD signals indicating that magnetism is not substantially suppressed at the interfaces. XMCD hysteresis loops measured at 2 K with magnetic field aligned in the [100] in plane direction showed similar values of the saturation magnetization. The reduction of the coercivity when the BTO layer is inserted (approaching the values characteristic of LSMO single films indicate a change of the magnetic anisotropy triggered by the iridate layer reported previously [5]. Magnetization vs temperature curves measured cooling in a 1000 Oe magnetic field showed also similar Curie temperatures for both samples, close to the values of single manganite layers.

Notice that the small cooling field of 1000 Oe is enough to reach very similar low temperature magnetization in both samples (See Supplementary Figure 6). This rules out non collinear magnetism (and also skyrmions) whose presence typically yields substantially increased saturation fields.

The very similar saturation magnetizations of bilayer samples with and without $BaTiO_3$ spacer indicate that if there is depressed magnetization of the LMSO at the SIO interface it has to be over very short length scale (of the order of 1 nm or less from the comparison of the saturation moments). Since LSMO shows relatively small values of the AHE ($\sigma_{xy}^{\mathrm{AHE}} = 6.54\ (\Omega cm)^{-1}$)

compared to SIO at the interface ($\sigma_{xy,I}^{\mathrm{AHE}} = -63\ (\Omega cm)^{-1}$), depressed magnetization of the LSMO does not explain the observed suppression of the AHE of the LSMO/SIO bilayers.

Magnetic measurements taken in samples with thinner 5 nm LSMO in a SIO/LSMO bilayer (See Supplementary Figure 7) still show square hysteresis loops, further supporting the absence of non collinear spin textures at the LSMO interface. Notice the 0.1T saturation field when magnetic field is applied along the [110] direction.

Because of the SrO double layer at the interface, the magnetic coupling between LSMO and SIO is much weaker than in the conditions considered in the previous theoretical work from three of the authors, which predicted topological Hall effect. The interface reconstruction, with double SrO planes, weakens the magnetic interaction and breaks the interfacial Dzyaloshinskii-Moriya interaction, and thus the canting giving rise to the spin textures responsible of the THE is not expected. Nevertheless, we cannot discard that the weak magnetic coupling between LSMO and SIO existing in our samples be a necessary ingredient for the emergence of magnetism in SIO.

**Supplementary note 5. Longitudinal Resistivity of $La_{0.7}Sr_{0.3}MnO_3$//$SrTiO_3$ and $SrIrO_3$//$SrTiO_3$ films.**

The longitudinal resistivity $\rho_{xx}$ for $La_{0.7}Sr_{0.3}MnO_3$//$SrTiO_3$ and $SrIrO_3$//$SrTiO_3$ ((See Supplementary Figure 8) have been measured in individual films at $T$ = 100 K, and found to be dependent on the thickness (respectively $d_{\mathrm{L}}$, and $d_{\mathrm{S}}$) only below ~4-5 nm.

**Supplementary note 6. Analysis of band dispersion and Berry curvature**

To gain insight into the origin of the large anomalous Hall conductivity we plot band dispersion relations near the Fermi level, 2D Fermi surfaces, and the separation between two adjacent bands, as well as Berry curvature for different cases in Supplementary Figure 9. Nodal lines existing in non-magnetic $SrIrO_3$ are unstable with respect to the magnetism, i.e. nonzero magnetic moment eliminates the nodal line and opens a gap. With the magnetic moment increases, different bands move up or down, creating many different kinds of band crossings. The plots show that such band crossing indeed produces the enhancement in the Berry curvature.

On each panel, top left figure shows the band dispersion, top middle 2D Fermi surface, top right 2D Berry curvature on the ($k_x$,$k_y$) plane. Bottom figures show the distance between two adjacent bands. Band crossing exists where the band separation is minimized. The strongest contribution to the Berry curvature comes from such band crossing at the Fermi level. This is indicated by the overlap of two Fermi surfaces as indicated by red circles in the 2D Fermi surface plot.

REFERENCES

[1] Momma K. and Izumi F., J. Appl. Crystallogr. VESTA3 for three-dimensional visualization of crystal, volumetric and morphology data, **44**, 1272– 1276 (2011)

[2] Xu W. J., Zhang B., Liu Z. X., Z. Wang Z., Li W., Wu Z. B., Yu R. H. and Zhang X. X. Anomalous Hall effect in Fe/Gd bilayers Eur. Phys. Lett. 90, 27004, (2010)

[3] Xu W. J., Zhang B., Wang Z., Chu S. S., Li W., Wu Z. B., Yu R. H. and Zhang X. X., Scaling law of anomalous Hall effect in Fe/Cu bilayers Eur. Phys. J. B, 65 (2008) 233 Eur. Phys. J. B 65, 233 (2008)

[4] Onoda, S., Sugimoto, N. & Nagaosa, N. Quantum transport theory of anomalous electric, thermoelectric, and thermal Hall effects in ferromagnets. *Phys. Rev. B* **77**, 165103 (2008)

[5] Yia, D. *et al.* Atomic-scale control of magnetic anisotropy via novel spin-orbit coupling effect in La2/3Sr1/3MnO3/SrIrO3 superlattices. *Proc. Natl. Acad. Sci. U. S. A.* **113**, 6397–6402 (2016).
